# Evolutionary conservation—evaluating the adaptive potential of species

**DOI:** 10.1111/eva.12227

**Published:** 2014-10-29

**Authors:** Christophe Eizaguirre, Miguel Baltazar-Soares

**Affiliations:** 1 School of Biological and Chemical Sciences, Queen Mary University of LondonLondon, UK; 2 Department of Evolutionary Ecology of Marine Fishes, GEOMAR Helmholtz Centre for Ocean ResearchKiel, Germany

**Keywords:** endangered species, evolutionary potential, genetic diversity

## Abstract

Despite intense efforts, biodiversity around the globe continues to decrease. To cease this phenomenon, we urgently need a better knowledge not only of the true extent of biodiversity, but also of the evolutionary potential of species to respond to environmental change. These aims are the heart of the developing field of *Evolutionary conservation*. Here, after describing problems associated with implementing evolutionary perspectives into management, we outline how evolutionary principles can contribute to efficient conservation programmes. We then introduce articles from this special issue on *Evolutionary conservation,* outlining how each study or review provides tools and concepts to contribute to efficient management of species or populations. Ultimately, we highlight what we believe can be future research avenues for evolutionary conservation.

Present days are often referred to as the 6th event of mass extinction (Leakey and Lewin 1996) because of human activities rapidly impacting biodiversity. This single fact calls for an urgent need for establishing efficient conservation programs. While ‘conservation’ and ‘evolutionary’ biology may appear to be opposing fields, it has been recognized that considering the adaptive potential of species improves the effectiveness of conservation practices. Here, we define the adaptive potential as the ability of species/populations to respond to selection by means of phenotypic or molecular changes. Despite accepting that species are not fixed entities, evolutionary processes are often overlooked by biologists and decision makers interested in protecting endangered species (Smith and Bernatchez 2008; Hendry et al. 2010; Moritz and Potter 2013). Of particular importance, are the high levels of inbreeding that small populations can be subjected to, which can increase homozygosity and the expression of deleterious recessive alleles (Charlesworth and Charlesworth 1987). In addition, small populations also suffer a loss of allelic diversity at functional genes, which can compromise the ability of a population to adapt to new or changing environments (Soulé 1985). Under the perspective of global change causing selective pressures on wild populations, many populations/species will not be able to migrate to their preferred environmental optima and must therefore adapt *in situ* to avoid extinction. Hence, it is imperative that the evolutionary processes driving species’ evolution are revealed to determine critical thresholds that reduce the persistence of species, communities, and ecosystems. Altogether, the overarching pillar of the field of *Evolutionary Conservation* is to provide a quantitative understanding of the dynamics of the evolutionary potential of species.

The neglect of evolutionary processes in conservation is unwarranted given that ecological and evolutionary processes may act at overlapping time scales (Hairston et al. 2005; Pelletier et al. 2009; Becks and Agrawal 2010; Ellner et al. 2011; Eizaguirre et al. 2012). Current conservation approaches can thus focus on the processes underpinning the adaptive potential of species (Stiebens et al. 2013).

Until recently, quantifying the genetic component of a species’ adaptive potential was technologically limited for nonmodel species as is the case for endangered species. However, this has changed with approaches combining technological advances (e.g. next-generation sequencing [NGS] and increased computational power) and theoretical breakthroughs that allow scanning entire genomes and increasing levels of spatial and temporal complexity of populations. Even though we are now in a better position to identify the genetic basis of adaptation and the mechanisms of adaptive responses in the wild, it remains a challenge to go beyond descriptive measures of patterns of genetic variation. The rapid advances made in sequencing technology will surely benefit conservation biology; however, it is not always clear how and why we should upscale from ‘genetics’ to ‘genomics’. Furthermore, as readers will see in this special issue, many important questions still remain to be addressed independently of genomics. Hence, we hope that the results and methods described in this special issue will serve as a blueprint for future work in the novel field of *Evolutionary Conservation*.

This special issue began with a symposium at the 2013 European Society of Evolutionary Biology (ESEB) meeting in Lisbon, Portugal and represents a broad cross section of research into evolutionary conservation covering three main aspects: (i) identifying and monitoring genetic diversity, (ii) understanding consequences of mating system and sexual selection on the adaptive potential of species, and (iii) determining the role of species–species interactions in conservation. Even though those three aspects are covered in this special issue, more exist and deserve attention as we highlight in what we consider should be future research avenues.

## Identifying and monitoring genetic diversity

While biodiversity contributes to the maintenance of ecosystems’ integrity (Hillebrand and Matthiessen 2009), the contribution of genetic diversity ranges from individual fitness, species’ evolutionary potential, and ecosystem stability. This observation directly poses the role of genetic diversity as crucial for species’ viability (Frankham et al. 2002). But how can we monitor genetic diversity? A usual perspective that has been undertaken by ecologists and evolutionary biologists is to monitor their species/population of interest over time. Temporal monitoring of the genetic status can inform policy and management actions mainly when major changes are being observed. However, by anticipation, establishing an appropriate monitoring scheme is crucial: which genetic metrics, temporal sampling protocols, and genetic markers are sufficiently sensitive and robust to be informative on conservation-relevant timescales? This question is indeed at the core of the work presented by Hoban et al. (2014) who utilized individual-based simulations. Key results address directly this question that many of us have faced when discussion with decision makers. The authors identified that sampling 50 individuals at two time points with 20 microsatellites could detect genetic erosion while 80–90% of diversity remained. Noteworthy, power increased substantially with more samples or markers. Furthermore, results suggest high power for studies using historic collections in monitoring program to compare past and contemporary fluctuations of genetic diversity.

Spurgin et al. (2014) recognized this strength and reconstructed the population history of the Seychelles warbler (*Acrocephalus sechellensis*) which, half a century ago, reached alarming low population size bringing the species close to extinction. Using DNA samples from contemporary wild populations and from museum specimens, their study spans 140 years. They showed 25% reduction in genetic diversity as well as signatures of bottleneck, with an effective population size falling from thousand to <50 within the last century. This kind of demographic reconstruction allows to better understanding patterns of genetic diversity, inbreeding, and promiscuity in the contemporary populations (Spurgin et al. 2014).

While understanding the past historic changes populations have experienced is important, trying to predict their capacity to respond ongoing pressures is crucial. It is known, for instance, that pollution—in particular high levels of synthetic estrogen (EE2) in water—affects many fish species at various developmental stages. Testing whether Alpine whitefish species carried the necessary genetic variation to adaptively respond to this new selection pressure, Brazzola et al. (2014) conducted full-factorial designs for each species. They revealed that despite toxic effects of the EE2 both species demonstrated the necessary additive genetic variation for an evolutionary response to this type of pollution. This study highlights how experiments can contribute to the characterization of a species evolutionary potential but also demonstrates that responses can be brought forward without large genomic screen.

Therefore, why should evolutionary conservation enter the ‘genomic world’ and how can we benefit from it? On the one hand, the major promise of genomics for conservation is the capacity to identify relevant functional diversity which allows species to thrive in their local environment (McMahon et al. 2014). A sufficient sample size and coverage will permit utilizing genome scans and identify genomic islands of selection. With sufficient resolution, genes can nowadays be identified. On the other hand, identifying cryptic population structure, important for the population functioning, may benefit from a genomic approach. Cryptic mechanisms entail local adaptation with gene flow (Stiebens et al. 2013; McMahon et al. 2014), gene flow from unidentified source population or cryptic pre- or postcopulatory mechanisms. All those factors affecting connectivity and reproduction are crucial parameters which deserve attention from a conservation point of view. Most importantly, however, integrating genome-wide diversity into conservation programs would avoid what McMahon and colleagues call the ‘emergency room conservation’ where considerable means would be needed to safe the species of interest.

Even though those promises are attractive, there are limits: (i) many traits are polygenic and can be under the control of many genes with minor relative effects, rendering the establishment of conservation measure solely based on genomics a complex task (Harrisson et al. 2014). Regulatory elements under epigenetic control may also be missed (Harrisson et al. 2014) and (ii) demographic events such as sudden bottlenecks can leave genomic signatures similar to those of selection—hence, appropriate knowledge of population history and demography is paramount (Spurgin et al. 2014). A functional guide for genome sequencing planning, as well as a step-by-step approach from sequencing to gene annotation, is also offered in this special issue (Ekblom and Wolf 2014). This will definitely facilitate the entry of conservation biologists into the field of evolutionary conservation. Lastly, (iii) phenotypic rescue (Chevin et al. 2013), where phenotypic plasticity buffers effects of strong selection, should not be neglected. Indeed, Brodersen and Seehausen (2014) argue in this special issue that monitoring programs which do not consider genetic diversity and phenotypic plasticity often fail to detect changes in these key components of biodiversity until after major losses of diversity have occurred. Even though focusing on fish, their suggestions go far beyond these taxonomic groups and can be extrapolated to all systems.

## Reproduction, sexual selection, and effective population size

One of the recognized problems in conservation biology is the effect of small population size on increasing risks of inbreeding and genetic drift affecting the adaptive potential of species. As previously pointed out, several factors ranging from current to historic population sizes can affect the magnitudes and directions of those effects. Experimental evolution tests revealed that fast inbreeding, due to small effective population size in *Drosophila*, results in large reduction in population mean fitness but, interestingly, populations with faster inbreeding expressed more heterosis upon interpopulation hybridization (Pekkala et al. 2014). This suggests the replenishment of genetic diversity benefits the population rapidly when it has been strongly compromised (Pekkala et al. 2014). Perrier et al. (2014) also demonstrate that alternative mating strategies in salmon increase effective population size and allelic richness – two major aspects of population viability. Clearly, such alternative mating strategies should be considered when designing stocking conservation programs or developing *ex situ* breeding designs. The later one is challenging because of all the above-mentioned genetic processes (i.e. inbreeding depression, random genetic drift) are also combined with selection and adaptation to captive environment which may then be traded-off for traits also important under wild conditions. Chargé et al. (2014) investigated this problem focusing on female mating strategy and how mate choice can influence captive breeding. The outcome of the review demonstrates that very few studies have considered the effects of captivity on sexual selection and the fitness costs associated. It is then obvious that accounting for female mate choice in captive breeding is in its infancy and many cryptic processes whether pre- or postcopulatory need to be investigated in the many species for which *ex situ* breeding programs are being designed (Chargé et al. 2014).

## Species–Species interaction and the adaptive potential

Clearly, there is growing interest in understanding how species–species interactions can affect the adaptive potential of the different partners. The classic example is the one of host–parasite interaction. Despite decades of research, we still lack knowledge on the ecological and genetic factors influencing the presence and severity of parasites. Focusing on the corncrake (*Crex crex*) which has a metapopulation system with reduced genetic structure but inhabits variable environments, Fourcade et al. (2014) evaluated the factors controlling the prevalence of haemosporidian parasites. Reduction in census population sizes, but not in genetic diversity, as well as anthropogenic activity has led to a reduction of host populations and pathogen prevalence. These results demonstrate that demographic and ecological factors can contribute to host–parasite interaction as much as genetic factors and confirm, once more, that there are important factors to be considered in conservation biology.

In Australia, viruses have been used as means to reduce the numbers of introduced rabbits which have a devastating impact on the native Australian environment. Studying the rabbit hemorrhagic disease virus (RHDV), Schwensow et al. (2014) show that in large interconnected metapopulations of rabbits, RHDV should maintain high virulence and cause short and strong disease outbreaks but it should also show low persistence in any given subpopulation. This new epidemiological framework is important for understanding virus–host coevolution and future disease management options of pest species to ensure persistence of native biodiversity.

Those two examples highlight the different perspectives on host–parasite interactions in management: on the one hand, we need to better understand the factors contributing to the spread of diseases, whether genetic, demographic, or ecological. This holds particularly true for endangered species exposed to emerging diseases. On the other hand, diseases (in the previous case, a virus) can also be a tool to regulate/manage invasive species which affect the ecosystem and further threaten local ecosystems’ integrity.

While host–parasite interactions have long been acknowledged as important evolutionary forces, molecular tools have revealed that more cryptic phenomena could also play important role. Hybridization is such a phenomenon and its outcome is unclear. Depending on unknown genetic factors but also on ecological niches and opportunities, hybridization can result in speciation (Nolte and Tautz 2011) or reverse speciation (Seehausen 2006). When this process is natural, research should focus on predicting its outcome. Human-induced hybridization of historically isolated taxa, however, raises conservation issues. The whitefish complex is an ideal model system to investigate porous reproductive isolation between otherwise geographically isolated evolutionary significant units. Even though clear genetic and phenotypic differentiation confirmed the endangered North Sea houting as an evolutionarily significant unit, admixture analyses revealed an extensive hybrid zone between North Sea houting, European whitefish, and Baltic houting (Dierking et al. 2014). Introgressive hybridization positively correlated with genetic diversity and was reflected in the adaptive traits such as gill raker counts. Testing possible causes of this hybridization pattern, the authors identified human stocking mistakes as primary drivers. Determining the outcome of hybridization with a combination of ecological characterization of the hybrid as well as an evaluation of their adaptive potential is the next research steps which should help informing managers on the viability of the endangered North Sea houting populations.

Altogether, examples of this special issue show that species’ interactions underlie evolutionary and ecological principles. They form pillars of ecosystem functioning and species structure (Clare 2014). Hence, understanding their structural mechanisms is crucial to predicting response to disturbance whether linked to invasive competitors, parasites, or change in environmental conditions. An accurate account of how species interact within their environment is fundamental to the establishment of good conservation practice in both a theoretical context, and in applied practice, for example, managing reintroductions and long-term monitoring. Those conceptual perspectives and how to develop food web analyses as toolkit to enter policy making process are developed in this special issue (Clare 2014).

## Future perspectives

With this special issue devoted to *Evolutionary Conservation*, it is clear that despite many advances and promises, our current weak understanding of the evolutionary potential of species has been hindered by numerous aspects. One major weakness is that experiments can hardly be conducted with endangered species. As a consequence, there is an urgent need to perform multigeneration population-based selection experiments with model species to tackle conservation-relevant questions. Secondly, from this special issue, it emerges that genomics can help us evaluating the functional genetic diversity relevant for species viability, but this cannot be achieved without (i) identifying the selective pressures whether linked to natural or sexual selection and (ii) solving the demographic history of the species. Lastly, to date the evaluation of the genetic diversity present in wild populations has neglected the phenotypic diversity and its nongenetic inheritance. Worse, it seems that there have been few, if any, attempts to dissect the relative contributions of genetic and epigenetic changes to the adaptive process in the context of endangered species. Identifying genomic regions responsible for adaptation or particularly prone to adaptive epigenetic changes provides new possibility for transferring lab resources to wildlife science. Filling those major knowledge gaps and combining them with ecological characterization of the species will bring new insights into the ways conservation programs can be designed.
